# Fluctuating Warm and Humid Conditions Differentially Impact Immunity and Development in the Malaria Vector *Anopheles stephensi*

**DOI:** 10.1111/gcb.70382

**Published:** 2025-08-05

**Authors:** Thais Lemos‐Silva, Emma De Neef, Yarno Valgaerts, Maria L. Simões

**Affiliations:** ^1^ Department of Biomedical Sciences Institute of Tropical Medicine Antwerp Antwerp Belgium

**Keywords:** *Anopheles stephensi*, bacterial infection, climate change, development & survival, diurnal fluctuation, environmental variability, innate immunity, malaria, temperature & relative humidity, vector competence

## Abstract

A variety of environmental factors including temperature and humidity influence mosquito physiology and behavior. However, their direct impact on innate immunity, the core mosquito defense system against pathogen infection, remains insufficiently understood. This is particularly important as climate change is likely already altering mosquito distribution. A key limitation in most mosquito studies is the use of unrealistic static temperature and humidity settings which fail to reflect natural environmental changes. To address this, we employed a novel approach, exposing *Anopheles stephensi* from egg to adult to diurnal fluctuations (+4°C and +10% relative humidity above the baseline). We then investigated the impact of these elevated fluctuating conditions on innate immune gene expression, development, and adult longevity. We show that realistic elevated temperature and humidity fluctuations prime basal immune responses and accelerate pre‐adult development without reducing adult lifespan. Bacterial infections under these elevated fluctuating conditions lead to a complex reprogramming of mosquito innate immunity and enhanced survival of both larvae and adults. Furthermore, fluctuating elevated temperature and humidity alter the transcriptional activity of key promoters widely used to express transgenes in genetically modified mosquitoes, highlighting the potential environmental sensitivity of these malaria control strategies. These results suggest that while elevated conditions‐driven immune priming could initially decrease *A. stephensi*'s vectorial capacity, the observed post‐bacterial challenge immune suppression could enhance susceptibility to *Plasmodium*, with potential significant implications for vector competence and malaria transmission. Our study findings highlight the need to incorporate realistic environmental variability in mosquito research to accurately predict the impact of climate on disease transmission.

## Graphical abstract

This study shows that fluctuating warm and humid conditions impact immune defenses, accelerate development, enhance post‐infection survival, and alter gene expression in mosquitoes, with potentially significant implications for their ability to vector malaria. Our findings highlight the need to incorporate realistic environmental variability in mosquito research to accurately predict the impact of climate on disease transmission.

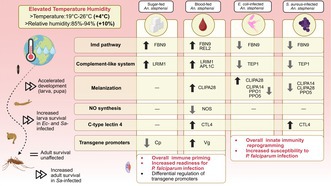

## 1 Introduction

Climate‐driven environmental alterations severely influence insect biology, impacting their physiology, metabolic activity, and distribution. Consequently, variations in environmental factors, such as temperature and humidity, alter mosquito susceptibility to infection and thus their ability to transmit pathogens (Beck‐Johnson et al. 2017; Brown et al. 2023; Mordecai et al. 2019; Paaijmans et al. 2009). This is a particularly important consideration for mosquito‐borne diseases such as malaria and for the African continent, where 95% of global malaria cases and deaths occur (World Health Organization (WHO) 2024), and which is experiencing increasing temperatures and shifting rainfall patterns that are likely exacerbated by climate change (World Meteorological Organization (WMO) 2024). Critically, climate change is presently thought to alter the geographic range of *Anopheles* species (Carlson et al. 2023; Kulkarni et al. 2016; Pinault and Hunter 2011). Moreover, *Plasmodium* infectivity and the extrinsic incubation period—the time the parasite requires to develop into an infectious stage that can be transmitted to humans—are also thermal‐sensitive, as we and others have demonstrated (Paaijmans et al. 2010, 2012; Pathak et al. 2019; Shapiro et al. 2017; Simões et al. 2022; Suh et al. 2020).

An increasing number of studies are trying to predict the effects of climate change on mosquito biology. However, most research on mosquitoes is conducted under fixed and optimal laboratory climatic settings (27°C temperature, 75% relative humidity (RH)). These conditions fail to mimic the natural environmental diurnal fluctuations that mosquitoes and pathogens experience in real‐world settings. For instance, the 2016–2025 average midday temperature in Addis Ababa, Ethiopia, on January 1 was 23°C, dropping to 9°C at midnight, with relative humidity inversely fluctuating from 30% to 75% (NASA Power 2025). Therefore, to effectively investigate the effect of climate change on mosquito biology, it is crucial to simulate these realistic daily temperature and humidity cycles in experimental settings.

The *Anopheles* innate immunity is its major defense system against invading microbes and a key determinant of the mosquito's ability to sustain human pathogen infection (vector competence). The activation of key anti‐microbial *Anopheles* signaling pathways is, to a significant degree, pathogen‐specific: the Toll pathway is typically triggered by Gram‐positive bacteria, fungi, and the rodent malaria parasite *Plasmodium berghei*, while the immune deficiency (Imd) pathway is triggered by both Gram‐positive and Gram‐negative bacteria and the human malaria parasite *Plasmodium falciparum*. NF‐κB transcription factors REL1 (Toll) and REL2 (Imd) translocate to the nucleus, activating the production of anti‐pathogen effectors, including antimicrobial peptides (AMPs), thioester‐containing proteins (TEPs), and fibrinogen‐related proteins (FREPs) (Clayton et al. 2014; Simões et al. 2018). Another key defense system that controls pathogen lysis and melanization in *Anopheles* is the complement‐like system, mediated by TEP1, LRIM1, and APL1C proteins (Baxter et al. 2010; Blandin et al. 2004; Fraiture et al. 2009; Povelones et al. 2009, 2011; Yassine et al. 2012).

Melanization, resulting in melanin production and pathogen encapsulation, is regulated by serine protease cascades and culminates in the activation of phenoloxidase enzymes (POs) (El Moussawi et al. 2009; Nakhleh, Christophides, et al. 2017; Nakhleh, El Moussawi, et al. 2017). In contrast, C‐type lectin 4 (CTL4) acts as a pathogen‐protective host factor by inhibiting melanization of *Plasmodium* and fungus (Osta et al. 2004; Simões et al. 2022; Simões, Mlambo, et al. 2017), making it a key candidate for further investigation into its role and impact on vector competence. Other important factors, such as the midgut enzyme nitric oxide synthase (NOS) participate in the mosquito's immune responses, leading to *Plasmodium* parasite and other pathogens' killing (Luckhart et al. 1998; Murdock et al. 2014; Peterson et al. 2007).

Despite increasing knowledge of *Anopheles*‐*Plasmodium* interactions and infection regulation mechanisms, malaria remains a major global health concern, with 597,000 deaths and 263 million cases reported in 2023—a higher number of cases compared to the previous year (World Health Organization (WHO) 2024). To help combat these devastating numbers, new vector‐based genetic strategies are rapidly progressing. These include developing genetically engineered *Anopheles* mosquitoes with higher *Plasmodium* resistance, aiming to replace natural malaria‐transmitting mosquito populations with *Plasmodium*‐refractory ones (Dong et al. 2022; Jones 2023; Simões 2023). Several transgenic *Anopheles* lines have been successfully engineered in the laboratory through the knockout (KO) of mosquito‐encoded *Plasmodium* host factors (agonists) or the overexpression of restriction factors (antagonists), including immune factors, under the regulation of appropriate promoters that enable the targeting of the malaria parasite in a relevant tissue and infection stage.

While *Anopheles* immune mechanisms and genetic control targets are increasingly understood, research on innate immunity, including studies focusing on identifying suitable genes for the engineering of genetically modified parasite‐refractory mosquitoes, is also typically performed under the described static and unrealistic climatic settings. The few existing studies investigating environmental effects on *Anopheles* immunity have focused on temperature with a limited number of genes only (Condé et al. 2021; Murdock et al. 2014, 2012), leaving the combined influence of temperature and humidity unexplored. In addition, in these studies, mosquitoes were exposed to different temperatures at the adult stage only.

To address these limitations, we developed a novel approach that incorporates diurnal environmental variability that is not random but instead is based on future climate change. These settings allow us to understand whether previous results yielded from constant conditions when comparing high and low temperature reflect a natural situation where environmental fluctuation occurs. We employed realistic experimental conditions where *Anopheles stephensi* mosquitoes were exposed from egg to adult stage to fluctuating elevated temperature and relative humidity (ETH) which simulated projected climate change conditions for Addis Ababa, Ethiopia, specifically +4°C and +10% RH relative to baseline. In this study, we aimed to gauge immediate *A. stephensi* immune and physiological responses to these fluctuating conditions, rather than performing genetic selection or investigating mosquito adaptation. This approach will contribute to predicting the effects of ETH on mosquito biology and to inform the experimental design of future research on climate change.

Our study not only addresses the escalating threats of climate change but also considers the recent rapid expansion of the primarily South‐Asian malaria vector *A. stephensi* into Africa. This expansion is thought to have already caused at least one malaria outbreak in Ethiopia (Emiru et al. 2023), one of the most vulnerable countries to climate change largely due to its economy and livelihoods relying heavily on agriculture—a sector extremely susceptible to climatic shifts—alongside its relatively low adaptive capacity (Abebe and Amare 2025; World Bank 2021). Given its established presence and potential for further spread within the country due to its ecological adaptability, including to urban environments (Taylor et al. 2024), *A. stephensi* serves as a highly relevant model for investigating the impacts of environmental variability. We demonstrate the impact of combined hourly variable elevated temperature and relative humidity on the *A. stephensi* basal and pathogen‐induced innate immune responses as well as on pre‐adult development and adult size and longevity. Furthermore, we investigate the environmental sensitivity of key promoters used to express transgenes in genetically modified *Anopheles* mosquitoes. This study provides crucial insights into the complex interplay between natural environmental variations and vectorial capacity, a mathematical approximation of the efficiency of vector‐borne disease transmission.

## 2 Materials and Methods

### 2.1 Mosquito Rearing and Experimental Design

An *A. stephensi* (SDA‐500 strain) colony was maintained under standard laboratory conditions (27°C and 75% RH) (see Data S1 for details). For each experiment, eggs collected from a cage with blood‐fed females were counted under a stereo microscope (Amscope) and separated in trays containing 200 eggs each. Trays were split in equal numbers between two cohorts/groups and placed in distinct environmental chambers (CPS‐P530, Rumed). A minimum of three trays with 200 eggs each were used per experiment and per group, each tray corresponding to a different biological replicate and a different generation.

### 2.2 Temperature and Humidity Regimes

To assess the effects of fluctuating temperature and humidity conditions on *A. stephensi*, we established two distinct temperature and humidity regimes based on current and future predicted climate conditions. To simulate a realistic current pattern of diurnal environmental fluctuations, a control group of mosquitoes was maintained throughout their life cycle (from eggs to adults) under 24‐h day/night temperature and relative humidity based on weather data for Addis Ababa, Ethiopia, for June 15, 2023 (CustomWeather, Inc n.d., accessed via Time and date 2023). While this humidity data was used as a basis, the relative humidity in the chambers could not go below 75% without dangerous larval water loss. The control group's environmental conditions, representative of the warm/wet season, were selected to avoid excessive cold or dryness that could undermine mosquito fitness.

A second group of mosquitoes, referred to as ETH conditioned mosquitoes in this study, was exposed to temperatures reflecting climate change projections for Ethiopia (SSP5‐8.5 scenario) of an approximated mean 4°C increase by 2080–2100 (World Bank 2024). Another simulation for the Awash river basin of Ethiopia projected an increase in maximum temperature of up to 4.1°C by the 2070s under a high‐emission scenario (Tadese et al. 2020). Although humidity projections for East Africa are weak and variable, the IPCC Sixth Assessment Report (Trisos et al. 2022) projected an increase in annual mean rainfall for the region, particularly in the eastern parts, at 2°C global warming. A 10% average increase in relative humidity compared to the control group was implemented for the ETH group throughout the day/night, informed by a recent study in a region in the Ethiopian highlands that projected a 7% increase in total annual precipitation by 2050 and a 17% increase during the rainy season (Rettie et al. 2022). In another study, the 2050s and 2070s medium‐emission simulations showed an increase in precipitation during half of the months, with 32% and 10%, respectively (Tadese et al. 2020). Therefore, the +10% RH selected for our study is a representative and conservative estimate within the projected ranges and served as an experimental representation of anticipated higher moisture levels potentially associated with increased precipitation in the region.

**FIGURE 1:**
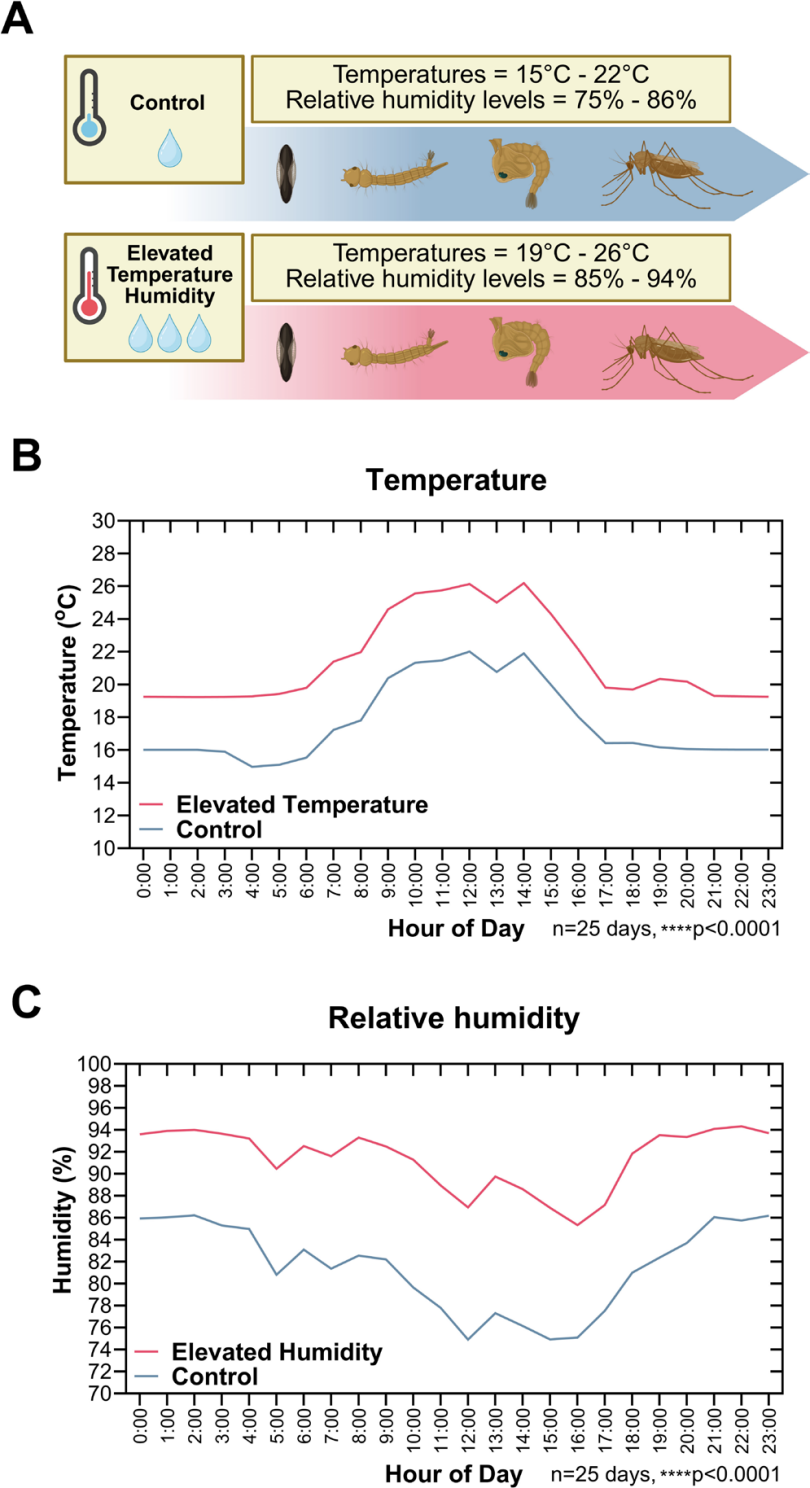
Overview of this study's experimental design. (A) Mosquitoes from the control group were exposed to hourly variable temperatures ranging from 15°C to 22°C and hourly variable relative humidity from 75% to 86% throughout their development (egg, larva, pupa, and adult). Mosquitoes from the elevated temperature and relative humidity (ETH) group were exposed to hourly variable temperatures 4°C higher (19°C–26°C) and hourly variable relative humidity 10% higher in average than the control group (85%–94%). Hourly temperature (B) and relative humidity (C) were measured inside the control (blue line) and ETH (red line) environmental chambers for 25 consecutive days and were compared. ****p < 0.0001.

The control and the ETH mosquitoes were maintained in distinct environmental chambers experiencing significantly different hourly variable temperature and relative humidity conditions throughout the day/night cycle (Table 1 (logged hourly), Figure 1, File S1). The average temperature in the control chamber fluctuated between 15°C and 22°C, while the ETH chamber maintained higher average temperatures between 19°C and 26°C (*p* < 0.0001). Similarly, control mosquitoes were maintained under average relative humidity between 75% and 86%, while ETH mosquitoes experienced relative humidity between 85% and 94% (*p* < 0.0001). Both cohorts were kept on a 12‐h day/night cycle (referring to the 12 h of light, and the 12 h of darkness within this cycle). These environmental conditions for the control and the ETH mosquitoes were used throughout the study and were continuously monitored using data loggers (M2‐TH, tempmate).

**TABLE 1:** Average hourly temperature and relative humidity employed in this study.

Time (hr:min)	Temperature (^o^C ave., difference)			Relative humidity (% ave., difference)		
	Control	ETH	ETH‐Control	Control	ETH	ETH‐Control
0:00	16.0	19.3	3.3	85.9	93.6	7.7
1:00	16.0	19.2	3.2	86.0	93.9	7.9
2:00	16.0	19.2	3.2	86.2	94.0	7.8
3:00	15.9	19.2	3.3	85.3	93.6	8.3
4:00	15.0	19.3	4.3	85.0	93.2	8.2
5:00	15.1	19.4	4.3	80.8	90.5	9.7
6:00	15.5	19.8	4.3	83.1	92.5	9.4
7:00	17.2	21.4	4.2	81.4	91.6	10.2
8:00	17.8	22.0	4.2	82.6	93.3	10.7
9:00	20.4	24.6	4.2	82.2	92.5	10.3
10:00	21.3	25.6	4.3	79.7	91.3	11.6
11:00	21.5	25.8	4.3	77.8	89.0	11.2
12:00	22.0	26.1	4.1	74.9	86.9	12.0
13:00	20.8	25.0	4.2	77.3	89.7	12.4
14:00	21.9	26.2	4.3	76.2	88.6	12.4
15:00	20.0	24.3	4.3	74.9	86.9	12.0
16:00	18.0	22.1	4.1	75.1	85.3	10.2
17:00	16.4	19.8	3.4	77.5	87.2	9.7
18:00	16.4	19.7	3.3	81.0	91.8	10.8
19:00	16.2	20.3	4.1	82.4	93.5	11.1
20:00	16.1	20.2	4.1	83.7	93.4	9.7
21:00	16.0	19.3	3.3	86.1	94.1	8.0
22:00	16.0	19.3	3.3	85.8	94.3	8.5
23:00	16.0	19.3	3.3	86.2	93.7	7.5
	Ave difference		3.9	Ave difference		9.9

*Note:* Fluctuating control (blue); fluctuating elevated temperature and humidity (red).

### 2.3 Pre‐Adult Development and Adult Female Longevity and Size Studies

Eggs from both fluctuating control and ETH groups were left to hatch, and the number of first‐instar (L1) larvae was counted and removed daily to determine the egg hatch rate. For each condition (control and ETH), at least 10 age‐matched larvae from the same generation were examined using an optical microscope (Olympus) at 3 days post‐hatch, and images were captured. Larvae were maintained from first‐ to fourth‐instars (L1–L4) until pupation. The number of pupae present each day in both control and ETH trays was recorded to evaluate the pupation development time and rate (Dong et al. 2018), with pupae mortality assessed. Pupae were retained until adult emergence, and the emergence rate was recorded for each control and ETH cohort during five consecutive days, with no pupa emerging beyond day 5.

To assess the lifespan of adult female mosquitoes reared under distinct environmental conditions, approximately 35 1‐day‐old adult females from each group were provided a 10% sucrose solution meal ad libitum immediately upon emergence and maintained in small cages (175 × 175 × 175 mm) under control and ETH conditions until their death. Mortality was recorded daily. To estimate differences in the size of control and ETH mosquitoes, at least 20 age‐matched female mosquitoes from the same generation were examined. Wing lengths were measured from the distal end of the alula to the tip of the wing of 1–4‐day‐old females (Dong et al. 2018) and images were analyzed using an optical microscope and ImageJ software. Each experiment was conducted with a minimum of three independent biological replicates; each replicate corresponded to a control and an ETH tray containing 200 eggs each.

### 2.4 Bacteria Challenge and Enumeration Studies

Bacterial infections were induced by thoracic pricking with *Escherichia coli* and *Staphylococcus aureus* as in Dimopoulos et al. (1997); He et al. (2017); Schnitger et al. (2007, 2009). A sterile PBS solution was used as a negative control (see Data S1 for details). For longevity assays, 20 L3–L4 larvae or 40 3‐ to 4‐day‐old adult female mosquitoes per group were infected with bacteria or pricked with PBS. Two hours post‐pricking, dead larvae and adult individuals were removed, and survivors were counted. Challenged larvae and adults were maintained under control or ETH conditions, and mortality assessments were performed over an 8‐day period post‐challenge, with dead individuals removed daily. To measure adult emergence, a subset of larvae was challenged with bacteria or PBS and processed as described until adult emergence was measured. Three independent biological replicates were performed for each experiment.

### 2.5 Tissue Collection

Newly emerged female mosquitoes from both the control and the ETH cohorts of the same generation were divided into two groups. One group was kept on a 10% sucrose solution for 3 days following adult emergence. The other group was kept on a 10% sucrose solution until provided a naïve blood meal 2 days after emergence. Approximately 2–3 h post‐feeding, females were briefly immobilized by chilling on ice and live sorted. Only visible engorged females were kept (on a 10% sucrose solution until dissection) and unfed ones were removed. Three days after emergence (corresponding to 24 h post‐blood feeding for the blood‐fed females), 20 midguts and 10 fat bodies from both sugar‐fed and blood‐fed *A. stephensi* females were dissected in PBS and collected into 1.5 mL tubes containing RLT buffer (RNeasy Kit, Qiagen). For tissue collection from challenged mosquitoes, age‐matched control and ETH larvae (L3–L4) and adults of the same generation were challenged with PBS or bacteria as described and collected 4 h post‐pricking for RNA extraction. Each experiment was conducted with a minimum of three independent biological replicates.

### 2.6 RNA Extraction and Real‐Time qRT‐PCR Analysis

Total RNA was isolated (RNeasy kit, Qiagen) from the midguts and fat bodies of non‐challenged control and ETH mosquitoes, or from larvae‐ or adult‐challenged whole mosquitoes from both groups. cDNA was synthesized (see Data S1 for details on cDNA synthesis and quantitative real time polymerase chain reaction (qRT‐PCR) protocols). The ‐fold change in gene expression was calculated according to the standard 2^−ΔΔCT^ method. The sequences of primers used for amplification can be found in Table S1.

### 2.7 Statistical Analysis

**FIGURE 2:**
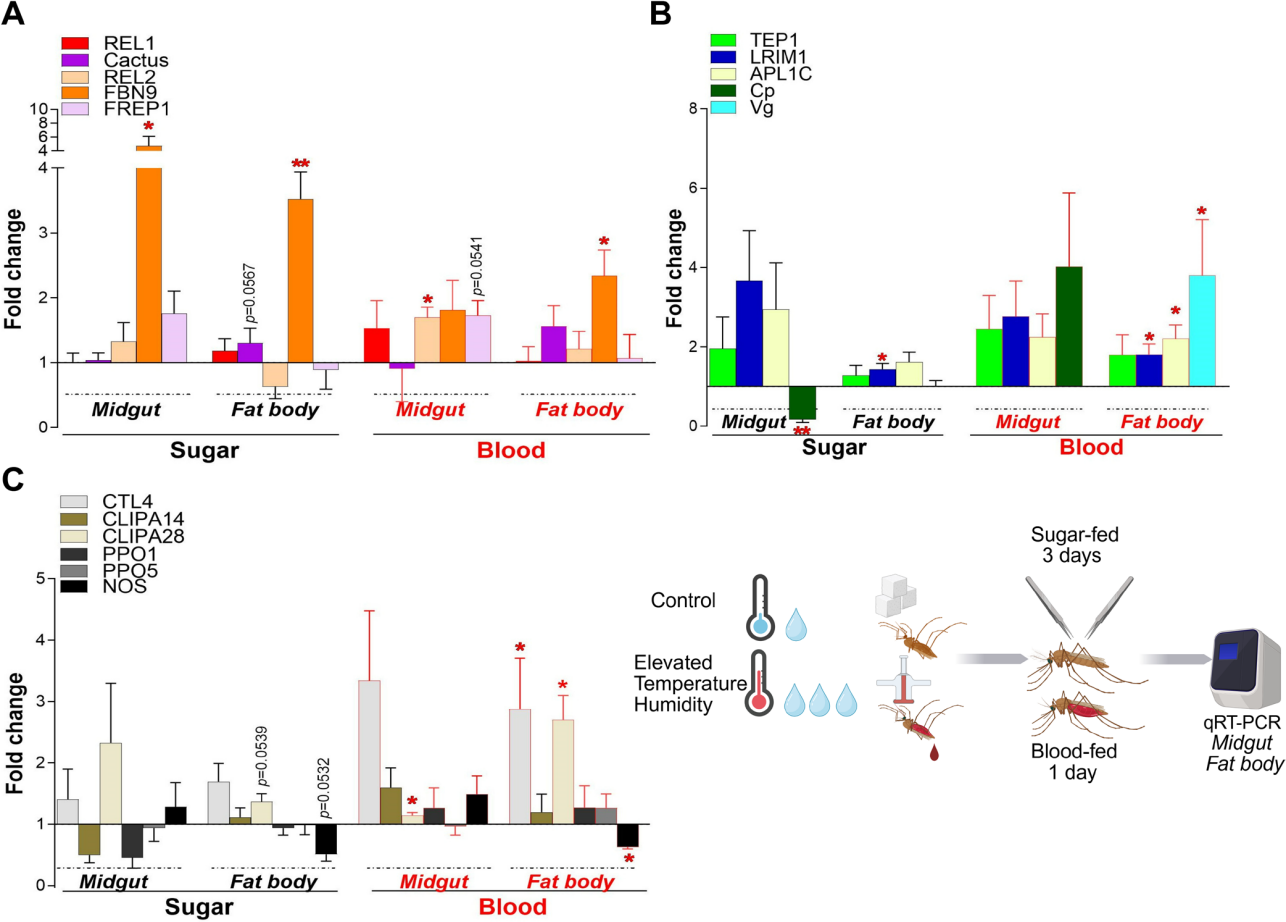
Influence of fluctuating elevated temperature and humidity on tissue‐specific immune priming in sugar‐fed and blood‐fed *Anopheles stephensi*. Basal transcriptional expression was assessed in the midgut and fat body‐containing carcass of sugar‐fed (3‐days post‐emergence) or blood‐fed (1‐day post‐blood feeding) *A. stephensi* female mosquitoes reared under ETH conditions and compared with mosquitoes reared under control conditions. Relative gene expression fold change was measured by qRT‐PCR for (A) Toll and Imd pathway key immune effectors, (B) complement‐like immune factors and promoters used to drive transgenes, and (C) melanization pathway‐related immune factors and nitric oxide synthase, and calculated using the 2^−ΔΔCT^ method. The line at *y* = 1 represents no change in gene expression under ETH conditions compared to control conditions. Bars above *y* = 1 indicate gene upregulation under ETH conditions; bars below *y* = 1 indicate gene downregulation under ETH conditions. Data represents mean and standard deviation (SD) of three to five independent biological replicates. Two‐tailed *p < 0.05; **p < 0.01.

**FIGURE 3:**
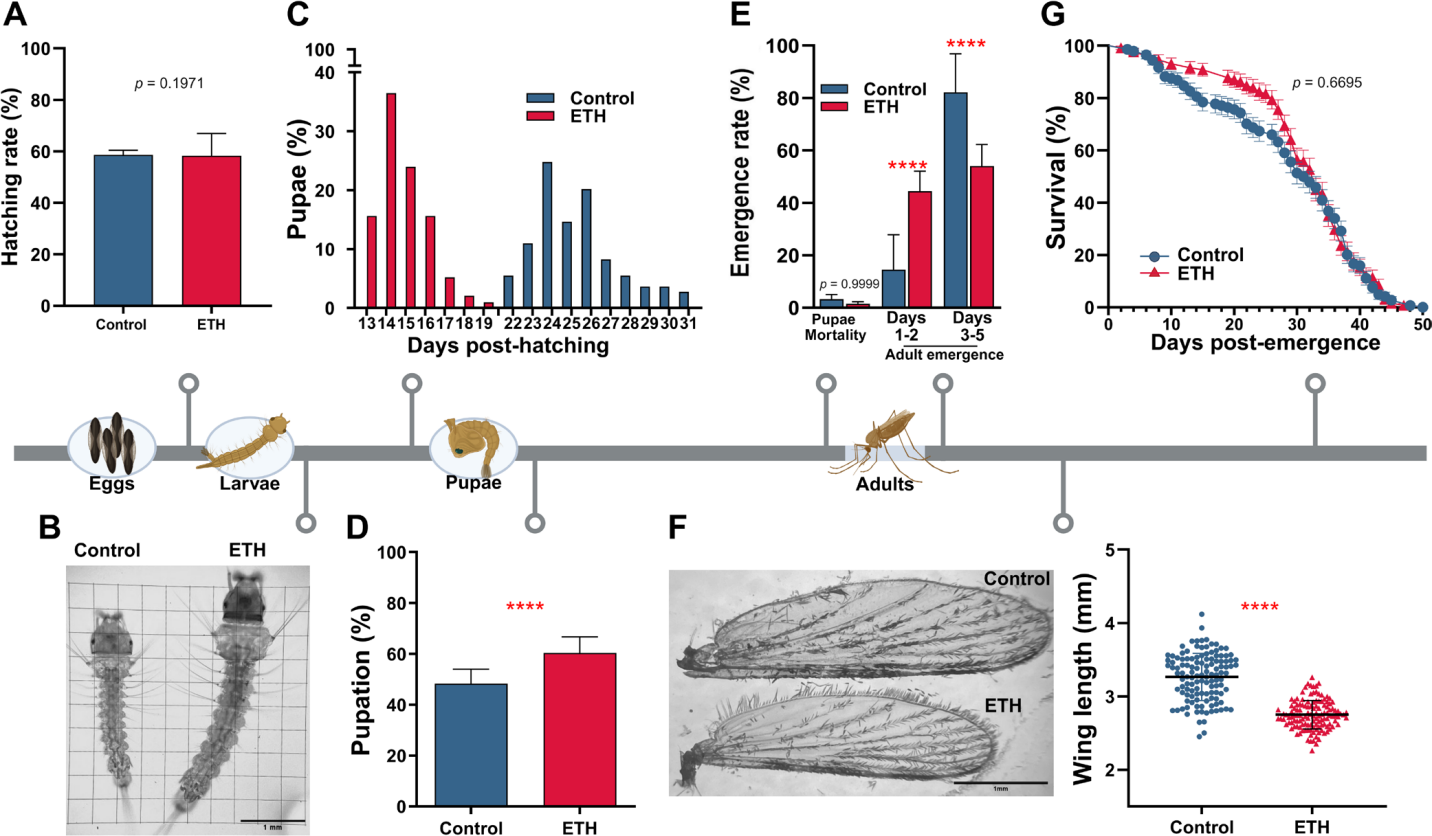
Impact of fluctuating elevated temperature and humidity on *Anopheles stephensi* pre‐adult development, and adult longevity and size. (A) Hatch rates indicate the average percentage of eggs giving rise to first instar larvae, as determined by three independent biological replicates. Mean values and standard errors (SE) are indicated. No statistically significant differences were observed. (B) Larvae reared under ETH conditions developed faster than those under control conditions, demonstrated by their larger size at 3 days post‐hatch. Scale bar 1 mm. (C) Pupation in the ETH group initiated earlier than in the control group. Median time to pupation for the ETH group was 14 days (interquartile range (IQR) 1 day), compared to 25 days for the control group (IQR 2 days). (D) Pupation rate under ETH conditions was significantly higher than under control conditions. Data represents mean values and SE of three independent biological replicates. ****p < 0.0001. (E) Mosquitoes from the ETH group emerged significantly earlier than those of the control group. Data represents mean values and SE of three independent biological replicates. ****p < 0.0001. (F) The wing lengths of ETH females were significantly shorter than those of the control females. Left panel: Representative images of wings from each group; scale bar 1 mm. Right panel: Each blue dot or red triangle represents an individual wing from the control or ETH groups, respectively. Data represents mean values and SD. Two‐tailed ****p < 0.0001. (G) Lifespan did not differ significantly between ETH and control mosquitoes. Data from four replicates is presented with standard error bars.

**FIGURE 4:**
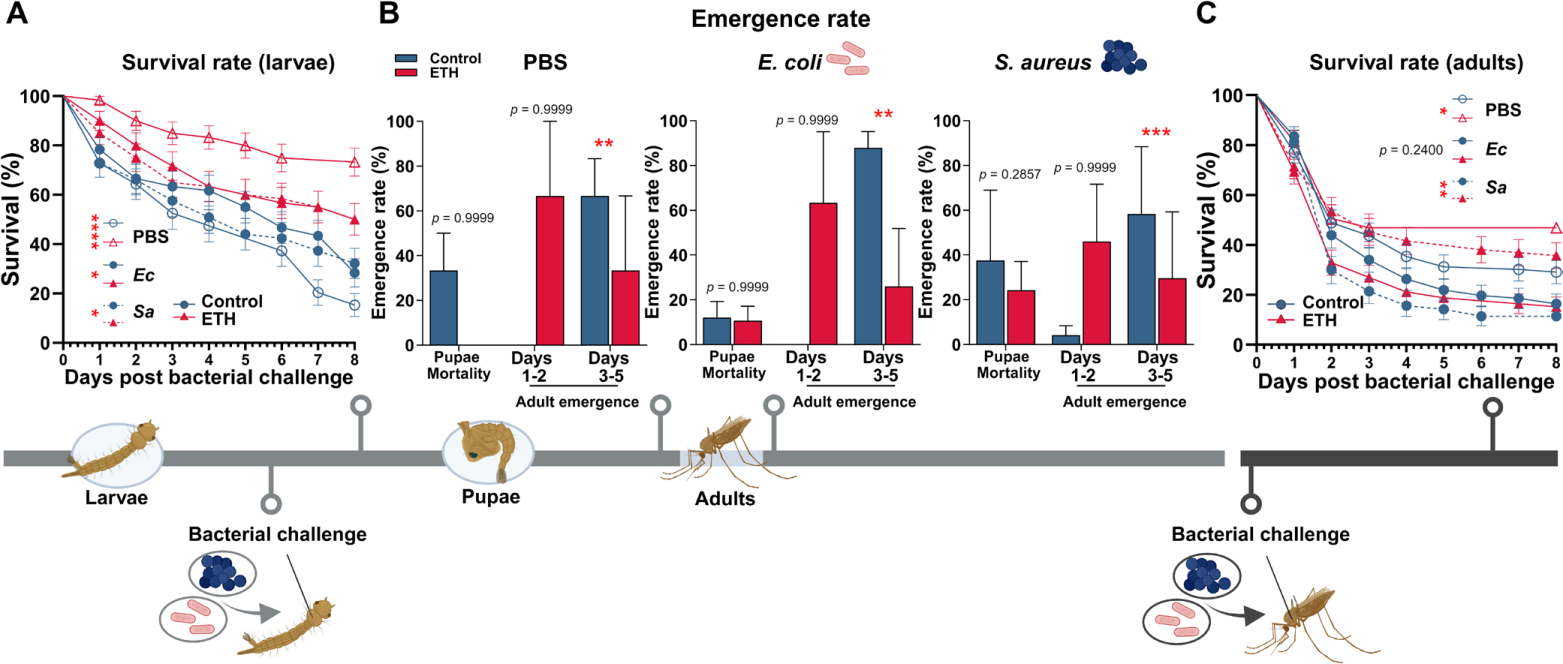
Impact of fluctuating elevated temperature and humidity on bacterial‐infected *Anopheles stephensi*. Larvae and female adult mosquitoes were challenged with phosphate‐buffered saline (PBS) as control, Gram‐negative bacterium *Escherichia coli* (*Ec*), or Gram‐positive bacterium *Staphylococcus aureus* (*Sa*). (A) Larvae from the ETH group showed significantly longer lifespan than larvae from the control group, for all challenges (PBS, *Ec*, *Sa*). Data from three independent biological replicates is presented with standard error bars. *p < 0.05; ****p < 0.0001. (B) Mosquitoes from the ETH group emerged earlier than those of the control group. Data represents mean values and SE of three independent biological replicates. **p < 0.01; ***p < 0.001. (C) The lifespan of adult female mosquitoes challenged with control‐PBS or *Sa* was significantly longer under ETH conditions compared to control conditions. Data from three independent biological replicates is presented with standard error bars. *p < 0.05; **p < 0.01.

**FIGURE 5:**
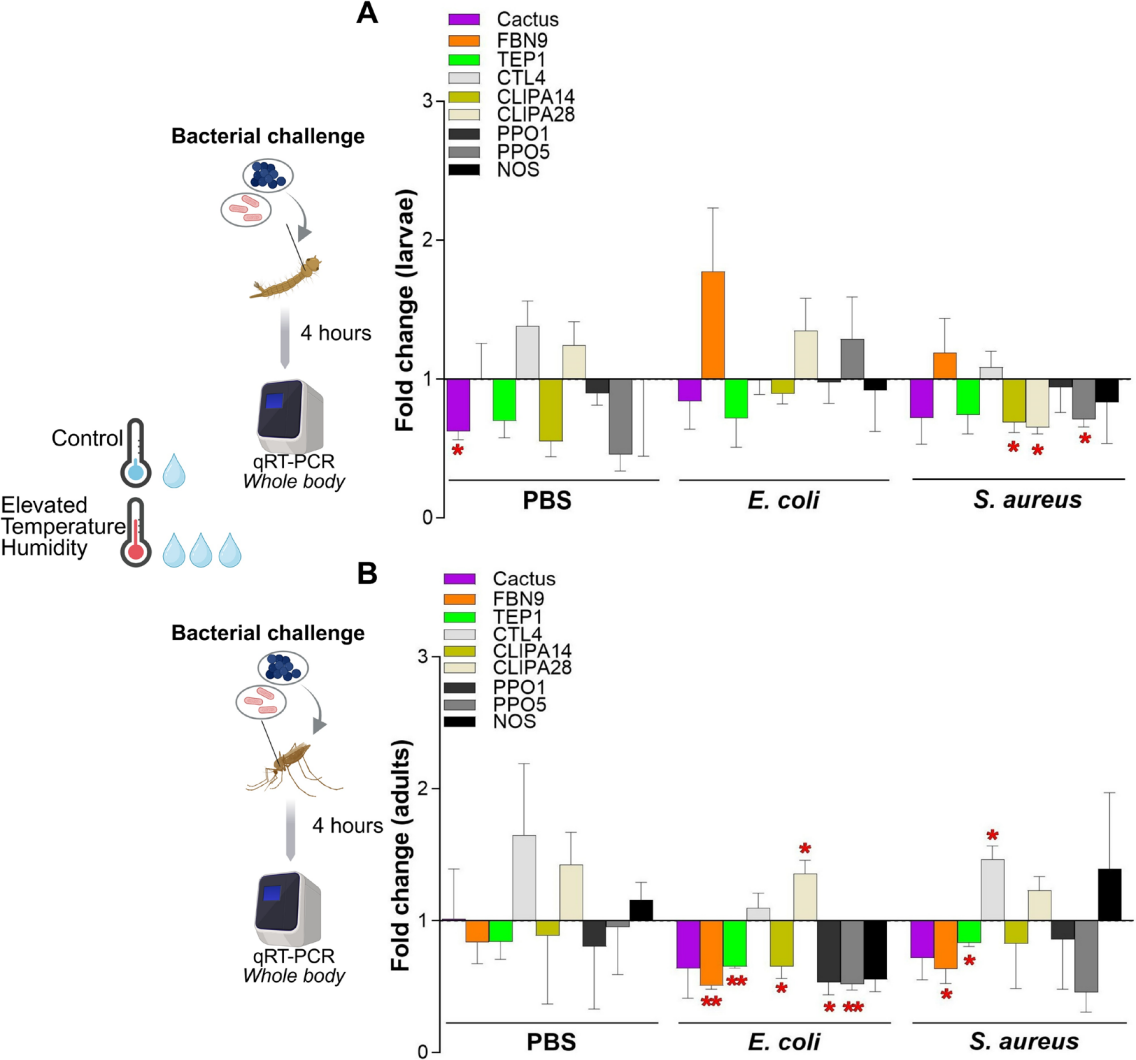
Reprogramming of innate immunity responses of infected *Anopheles stephensi* by elevated temperature and humidity. (A) Larvae and (B) female adult mosquitoes were challenged with phosphate‐buffered saline (PBS) as control, Gram‐negative bacterium *Escherichia coli* (*Ec*), or Gram‐positive bacterium *Staphylococcus aureus* (*Sa*). Gene expression was assessed at 4‐h post‐challenge in the whole body of *A. stephensi* larvae/adults reared under ETH conditions and compared with larvae/adults reared under control conditions. Relative gene expression fold change was measured by qRT‐PCR for Toll and Imd pathway key immune effectors, complement‐like immune factors, melanization pathway‐related immune factors and nitric oxide synthase, and calculated using the 2^−ΔΔCT^ method. The line at *y* = 1 represents no change in gene expression under ETH conditions compared to control conditions. Bars above *y* = 1 indicate gene upregulation under ETH conditions; bars below *y* = 1 indicate gene downregulation under ETH conditions. Data represents mean and SD of three to four independent biological replicates. Two‐tailed *p < 0.05; **p < 0.01.

Each experiment performed in this study compared the control and the ETH mosquitoes of the same generation; that is, mosquitoes derived from eggs laid by the same parental cohort. Each biological replicate corresponds to a different generation. All graphs were generated using GraphPad Prism10 software. Some figure panels were created with Biorender.com. Statistical analyses were performed as follows: Figure 1: hourly temperature and RH comparisons were performed using the *t*‐test with Bonferroni correction. Figures 2 and 5: Relative gene expression fold change was calculated using the 2^−ΔΔCT^ method. Comparisons were performed using two‐tailed *t*‐test. Figure 3A,D,E: Hatch, pupation, and emergence rates, and pupae mortality were analyzed using the Fisher's exact test. (C) Median time to pupation and interquartile ranges were calculated. (F) Wing length comparisons were performed using two‐tailed *t*‐test. (G) Lifespan was analyzed by Kaplan–Meier survival analysis with Mantel–Cox test. Figure 4A,C: Larval and adult survival were analyzed by Kaplan–Meier survival analysis with Mantel–Cox test. (B) Mosquito emergence rates and pupae mortality were analyzed using the Fisher's exact test. Details can be found in Files [Link], [Link], [Link].

## 3 Results and Discussion

### 3.1 Increased Temperature and Humidity Conditions Potentiate the Imd Pathway and Prime Mosquito Innate Immunity

To assess the combined impact of fluctuating elevated temperature and relative humidity on *A. stephensi* immunity, we measured the basal expression of key innate immunity genes in the midgut and fat body‐containing carcass of sugar‐fed and blood‐fed adult females using qRT‐PCR (Figure 2). Gene transcription reflects the implication of genes in physiological processes at the assayed conditions and can therefore be used to study the functional responses of an organism to climatic conditions (Aguilar et al. 2007). While most innate immunity studies have focused on the *Anopheles gambiae* mosquito, a conserved role is expected for immunity genes in the *A. stephensi* mosquito, given the high level of orthology between these two malaria vector species. First, we focused on key regulators of the Toll (REL1 is a positive regulator and Cactus is a negative regulator) and Imd (REL2 is a positive regulator) pathway‐mediated immune responses. While the expression of the Toll pathway's *REL1* and its negative regulator *Cactus* remained largely unaffected (despite a trend towards increased *Cactus* expression in the fat body‐containing carcass of non‐blood‐fed mosquitoes), *REL2* was expressed at significantly higher levels (1.7‐fold change, *p* = 0.0104) in the midgut of ETH females 24 h after blood feeding in comparison to females exposed to control climate conditions (Figure 2A), suggesting that increased temperature and relative humidity positively regulate Imd pathway activity. Next, we investigated the transcriptional expression of key *Anopheles* infection regulators from the FREP family: *FBN9* (fibrinogen domain containing immunolectin 9), which suppresses infection, and *Plasmodium* host factor *FREP1* (fibrinogen‐related protein 1), which facilitates midgut infection. FBN9, a potent anti‐*Plasmodium* immune factor that plays a role in anti‐bacterial defense as well (Christophides et al. 2002; Dong et al. 2006; Dong and Dimopoulos 2009), has been shown to exhibit higher basal expression in the fat body‐containing carcass than in the midgut of uninfected *A. gambiae* mosquitoes. Yet, FBN9 co‐localizes with *P. berghei and P. falciparum* ookinetes in infected midguts (Dong and Dimopoulos 2009). Our previous work demonstrated that transgenic FBN9 overexpression enhances *A. gambiae* resistance to *P. berghei* and bacterial infections (Simões, Dong, et al. 2017). *FBN9* basal expression was significantly increased in both the midgut (4.7‐fold change, *p* = 0.0323) and the fat body (3.5‐fold change, *p* = 0.0022) of sugar‐fed mosquitoes, and in the fat body (2.3‐fold change, *p* = 0.0414) 24 h after blood feeding under elevated temperature and relative humidity (Figure 2A). FREP1 acts as a *Plasmodium* agonist/host factor, integrating into the mosquito midgut's peritrophic matrix to facilitate parasite immune evasion; its knockout renders *A. gambiae* refractory to both *P. falciparum* and *P. berghei* (Dong et al. 2018; Zhang et al. 2015). In contrast to *FBN9*, *FREP1* expression showed no significant change in mosquitoes exposed to higher temperature and relative humidity compared to control mosquitoes, although a trend towards increased expression was observed in the midguts of blood‐fed females under these conditions (*p* = 0.0541) (Figure 2A).

These results demonstrate that fluctuating increased temperature and relative humidity significantly alter the basal expression of key *A. stephensi* encoded *Plasmodium* infection modulating genes. Specifically, the upregulation of *REL2* in the midgut of blood‐fed mosquitoes and *FBN9* in both tissues and across feeding states suggests a higher activity of the Imd pathway, a crucial component of the mosquito's defense against bacteria and *P. falciparum* infections. Further studies will address the effect of combined ETH on *P. falciparum* infection.

### 3.2 Elevated Temperature and Humidity Conditions Upregulate Anti‐*Plasmodium* Complement‐Like Factors

Considering the pivotal role of the complement‐like system in modulating *Anopheles* immunity to bacteria, fungi, and *Plasmodium*, we examined the expression of its core components under elevated temperature and relative humidity. LRIM1 and APL1C circulate in the mosquito hemolymph as a disulfide‐linked antagonist complex, which binds to mature TEP1, stabilizing it, ultimately activating pathogen lysis and melanization (Povelones et al. 2009, 2011). Under our ETH conditions, while *TEP1* mRNA abundance remained unchanged, we observed significant upregulation of *LRIM1* in the fat body‐containing carcass of both sugar‐fed (1.4‐fold change, *p* = 0.0356) and blood‐fed (1.8‐fold change, *p* = 0.0307) ETH mosquitoes compared to control mosquitoes. Similarly, *APL1C* expression increased significantly in the fat body of blood‐fed ETH mosquitoes at 24 h after blood feeding (2.2‐fold change, *p* = 0.0467) (Figure 2B).

These findings show that simulated ETH conditions significantly impact the *A. stephensi* complement‐like system, potentially affecting the mosquito's response to pathogens. It remains to be tested if the observed increased LRIM1/APL1C mRNA levels specifically translate to higher TEP1 cleavage or functional *Plasmodium* killing under ETH conditions. This is a critical direction for future research in our laboratory.

The broader increase in the expression of key *Anopheles* immune defense genes in different tissues under different feeding conditions supports our hypothesis of a heightened immune activity triggered by the stress of ETH. While this transcriptional upregulation potentially predisposes the mosquito to mount a stronger response to subsequent pathogen exposure, linking basal upregulation to functional killing remains an inference. An important consideration is the role of the mosquito's innate immune system in preventing the over‐proliferation of its natural microbiota mostly contained in the midgut (Saraiva et al. 2016). Indeed, our preliminary studies show that this microbiota proliferates to higher levels at fluctuating higher temperature and humidity conditions (Figure S1). It is therefore likely that some of the differential expressions of genes between the two compared climatic conditions reflect a greater need to control the midgut microbiota at ETH conditions.

### 3.3 Elevated Temperature and Humidity Conditions Influence the Expression of Promoters Used to Drive Anti‐*Plasmodium* Transgenes

Numerous *A. gambiae* and *A. stephensi* genetically engineered transgenic lines utilize the Carboxypeptidase (Cp) or the Vitellogenin (Vg) promoters to drive the expression of innate immunity genes or host factors in the midgut or the fat body‐containing carcass tissues, respectively, after a blood meal through which the malaria parasite is ingested. Examples of these genetically modified *Anopheles* lines include the *Plasmodium*‐resistant *A. stephensi CpREL2* and *VgREL2*, the *A. gambiae Vg‐TEP1r*, the *A. gambiae Vg‐FBN9*, and the *A. gambiae FREP1‐KO* (Dong et al. 2011, 2018; Simões, Dong, et al. 2017; Volohonsky et al. 2017). *Cp* showed a significantly lower expression in the midgut (0.2‐fold change, *p* = 0.0064) of sugar‐fed ETH mosquitoes (Figure 2B). Interestingly, *Vg* expression was significantly increased in the fat body of ETH mosquitoes 24 h after blood feeding (3.8‐fold change, *p* = 0.0414, Figure 2B), compared to control mosquitoes, indicating that the blood meal inducibility of this promoter was stronger under ETH conditions.

The observed impact of fluctuating ETH on the transcriptional levels of key promoters commonly used in transgenic *Plasmodium*‐resistant *Anopheles* may have important implications for the efficacy and design of vector‐based genetic strategies for malaria control. In particular, the altered transcriptional activity of the *Vg* promoter under higher temperature and humidity conditions demonstrates its environmental sensitivity. This can have implications for the effectiveness of a transgene driven by the Vg promoter, as its efficacy could vary significantly depending on the external environment. Our findings further highlight the need to use realistic environmental variations in the laboratory before field trials and incorporate promoters that are not strongly affected by climate change in future transgenic mosquito designs.

### 3.4 Fluctuating Elevated Temperature and Humidity Conditions Modulate the Expression of Melanization‐Regulating Factors and Suppress the Expression of Nitric Oxide Synthase‐Mediated Responses Upon Blood Feeding

In *A. gambiae*, rodent *P. berghei* parasites can become completely melanized by a complement‐like‐dependent mechanism, which they evade by exploiting the C‐type lectin agonists/host factors CTL4 and CTLMA2 (Osta et al. 2004). Our previous work using CTL4‐KO mosquitoes revealed that *A. gambiae* melanizes human *P. falciparum* in a process that is complement‐independent but temperature‐dependent (Simões et al. 2022). Here, we extend these findings by demonstrating that the combined influence of elevated temperature and relative humidity significantly increases *CTL4* expression (2.9‐fold change, *p* = 0.0500) in the fat body of blood‐fed ETH mosquitoes compared to control mosquitoes 24 h after blood feeding (Figure 2C). Melanin formation in the mosquito results from the proteolytic activation of PPOs to PO, induced by a cascade of CLIP serine proteases. Transcription of CLIP‐serine protease homolog 14 (*CLIPA14*), an agonist for *P. berghei*, *P. falciparum*, Gram‐negative, and Gram‐positive bacterial infections (Nakhleh, Christophides, et al. 2017; Simões et al. 2022), was not significantly affected by temperature and humidity variations (Figure 2C). In contrast, *CLIPA28*, which acts as a pathogen antagonist in the melanization of *P. berghei* ookinetes and bacteria (El Moussawi et al. 2009), was significantly higher expressed in the midgut (1.2‐fold change, *p* = 0.0485) and fat body‐containing carcass (2.7‐fold change, *p* = 0.0222) of blood‐fed ETH females compared to control females (Figure 2C). The transcription of *PPO1* has been shown to be significantly increased in the whole body of *A. albimanus* mosquitoes at 6 h after exposure to 37°C heat shock (Condé et al. 2021). Here, exposure to higher variable temperature and humidity levels did not affect the expression of prophenoloxidases *PPO1* nor *PPO5* (Figure 2C).

Nitric oxide synthesis in infected midguts generates toxic metabolites that inhibit *Plasmodium* development (Peterson et al. 2007). In the present study, we observed no significant changes in *NOS* expression in sugar‐fed mosquitoes, but significantly lower expression (0.6‐fold change, *p* = 0.0114) in the fat body‐containing carcass of blood‐fed mosquitoes exposed to ETH, compared to control mosquitoes (Figure 2C). Comparisons with previous studies are detailed in Data S1.

Our findings suggest a complex, likely antagonistic interaction between different immune pathways that potentially results in complex and opposing effects on mosquito readiness to encounter pathogens, including *Plasmodium*. While basal transcriptional upregulation of *CLIPA28* could lead to the activation of anti‐pathogen defenses, the observed *NOS* downregulation in the fat body of ETH blood‐fed mosquitoes could diminish their ability to effectively kill *Plasmodium* ookinetes during their maturation and invasion of the epithelium. In follow‐up studies, we will evaluate how fluctuating high temperature and humidity affect *P. falciparum* infection prevalence and intensity. Importantly, our results demonstrate that gene expression kinetics differ under realistic environmental variations compared to the traditionally used static environmental conditions. Our *NOS* expression assay highlights that the regulation of innate defenses previously linked to anti‐*Plasmodium* activity through standard fixed laboratory conditions may exhibit different expression kinetics and possibly different mechanisms of action under realistic fluctuating conditions.

The overall more pronounced differences in basal gene expression observed in blood‐fed compared to sugar‐fed mosquitoes between the control and ETH conditions can partly reflect an increase in hemocytes, a major source of immune factors, after the blood meal (Bryant and Michel 2014), as well as the midgut microbiota that proliferates to high levels in the nutrient‐rich blood (Saraiva et al. 2016). In addition, blood feeding also temporarily increases both internal temperature and water content (Benoit et al. 2011), which can further intensify the effects of the ETH conditions we used.

### 3.5 Combined Fluctuating Higher Temperature and Humidity Accelerate *A. stephensi* Development Without Affecting Adult Lifespan

To further elucidate how fluctuating ETH may affect mosquito vectorial capacity, we investigated the combined effects of hourly variable temperature and relative humidity on *A. stephensi* development across all life stages. Egg hatch rates were similar between the control and the ETH cohorts (Figure 3A), in accordance with prior studies with several *Anopheles* species at constant humidity and increased constant temperatures (Agyekum et al. 2021). However, larvae developed faster at our ETH conditions compared to those reared at control conditions, evidenced by their advanced stage at three days post‐hatching (Figure 3B) and earlier pupation (Figure 3C), in agreement with findings in *A. gambiae* and *A. stephensi* under constant humidity and temperatures below 30°C (Barreaux et al. 2018; Christiansen‐Jucht et al. 2015; Paaijmans et al. 2013; Tuno et al. 2023). The pupation rate was also significantly higher in the ETH group (*p* < 0.0001, Figure 3D), consistent with previous studies focusing on other mosquito species reared at constant humidity and increasing temperatures (Agyekum et al. 2022; Mamai et al. 2018). Adult female emergence also occurred significantly earlier at ETH conditions compared to control conditioned females (*p* < 0.0001, Figure 3E), reflecting the observed accelerated larval and pupal development. While this faster development resulted in smaller ETH adults, as measured by wing length (Figure 3F), it did not compromise their lifespan (Figure 3G) (detailed analysis and discussion in Data S1).

These results suggest that the fluctuating simulated ETH confers a fitness advantage to *A. stephensi* by accelerating pre‐adult development without compromising adult viability. Importantly, comparing our findings with those from studies using constant temperature and relative humidity highlights the need to use realistic, fluctuating conditions to accurately assess how climate impacts mosquito fitness. While we employed a realistic setup where temperature and relative humidity are varied simultaneously, it would be interesting to dissect the independent contribution of humidity to the observed developmental changes in future studies.

### 3.6 Fluctuating Elevated Temperature and Humidity Prolong Bacteria‐Challenged Larval Survival and Adult Resistance to *S. aureus*

Bacteria and fungi are ubiquitous pathogens encountered by mosquitoes in the field; hence, the mosquito immune system has mostly evolved to combat these commonly occurring microorganisms in their natural habitats (Simões and Dimopoulos 2015). Understanding how fluctuating temperature and humidity affect mosquito resistance to bacteria is highly relevant, as it could influence mosquito survival and susceptibility to *Plasmodium*, thus impacting the dynamics of malaria transmission. We monitored the survival of larvae and adult female control and ETH *A. stephensi* mosquitoes at 4 h following pricking with either PBS as control, Gram‐negative bacterium *Escherichia coli*, or Gram‐positive bacterium *Staphylococcus aureus* (Figure 4). Combined fluctuating ETH conditions were highly favorable for larval survival across all three challenged groups (Figure 4A). Details can be found in Data S1.

Despite our simulated ETH conditions having a positive effect on larval survival, they did not affect pupae mortality (Figure 4B). In addition, the acceleration of the adult emergence rate (Figure 4B) under ETH conditions was independent of the challenges (PBS, *E. coli* or *S. aureus* ) larvae were given, while ETH adult females challenged with PBS exhibited significantly higher survival (*p* = 0.0424) than control ones (Figure 4C). Comparisons with unchallenged mosquitoes are provided in Data S1. This suggests that fluctuating ETH may mitigate the stress associated with wounding, potentially by promoting faster healing of the injection wound. Interestingly, combined fluctuating temperature and humidity conferred a clear survival advantage to mosquitoes challenged with *S. aureus* (*p* = 0.0044) but did not significantly affect the survival of *E. coli* ‐challenged adults (Figure 4C).

Taken together, our results demonstrate that the impacts of elevated temperature and relative humidity on bacterial‐challenged *A. stephensi* mosquitoes vary with the mosquito developmental stage. Furthermore, the effects of simulated fluctuating ETH on adult survival are microbial challenge‐specific, suggesting that certain immune pathways and effectors may be differentially affected by these environmental changes. For example, combined ETH appear to enhance the stress mitigation response to *S. aureus* infection, providing adult mosquitoes with a protective effect against this bacterium (Figure 4C). Specific responses to *Plasmodium* remain to be tested.

### 3.7 Fluctuating Elevated Temperature and Humidity Reveal Novel Immune Gene Expression Patterns in Bacteria‐Challenged Mosquitoes

To investigate the impact of ETH conditions on mosquito early immune‐related responses after bacterial ( *E. coli* or *S. aureus* ) infection compared to injury (PBS‐challenge), we analyzed the expression of selected genes involved in both anti‐bacterial and anti‐*Plasmodium* defenses, in both larvae and adults (Figure 5). A 4‐h post‐challenge time point was selected to capture the initial transcriptional innate immune responses (Dimopoulos et al. 1997). Larvae exposed to higher fluctuating temperature and humidity showed significant downregulation of *Cactus* (0.6‐fold change, *p* = 0.0141) compared to control larvae at 4 h post‐PBS injury (Figure 5A), unlike *A. gambiae* larvae (League et al. 2017). *S. aureus* infection also significantly decreased the relative expression of melanization regulators *PPO5* (*p* = 0.0470), *CLIPA14* (*p* = 0.0443), and *CLIPA28* (*p* = 0.0293) in ETH larvae, while no differences in gene regulation were observed when larvae were infected with *E. coli* (Figure 5A).

Injury (PBS‐challenge) alone did not significantly alter gene expression of adult mosquitoes at 4 h post‐challenge at ETH conditions compared to control conditions (Figure 5B). However, bacterial challenges resulted in differential effects between the two climatic conditions. Specifically, challenge with *E. coli* resulted in a significantly lower expression of *FBN9* (0.5‐fold change, *p* = 0.0021) and *TEP1* (0.6‐fold change, *p* = 0.0015) in mosquitoes exposed to higher fluctuating temperature and relative humidity, compared to control mosquitoes (Figure 5B). *E. coli* infection resulted in lower expression of *PPO1* in ETH mosquitoes (*p* = 0.0201) compared to control mosquitoes (Figure 5B). Additionally, we observed significantly lower expression of the melanization regulators *PPO5* (*p* = 0.0047) and *CLIPA14* (*p* = 0.0435), while *CLIPA28* was expressed at higher levels (*p* = 0.0314) following *E. coli* challenge of ETH mosquitoes compared to control mosquitoes (Figure 5B). Decreases in PO activity have been observed in several insect species after heat exposure (League et al. 2017). Similarly to the response to *E. coli* , ETH adults challenged with *S. aureus* expressed significantly lower levels of *FBN9* (*p* = 0.0478) and *TEP1* (*p* = 0.0330) (Figure 5B), consistent with previous studies conducted at constant temperature and humidity (Dong et al. 2006; Dong and Dimopoulos 2009). Conversely, elevated temperature and relative humidity significantly increased the expression of *CTL4* in *S. aureus‐*challenged ETH mosquitoes (1.5‐fold change, *p* = 0.0108) compared to control ones (Figure 5B). This finding is particularly interesting, as we have previously reported that CTL4 is an antagonist of *E. coli* but not *S. aureus* infection, and its regulation of melanization is temperature‐dependent (Simões et al. 2022). Further discussion of the transcriptional profiles post‐challenge can be found in Data S1.

Our findings confirm that *Anopheles* immune responses are mosquito stage‐specific (League et al. 2017), pathogen‐dependent, and significantly impacted by fluctuating ETH conditions. The observed lower expression of key *Anopheles* innate immunity genes in adults infected with either bacterium at higher temperature and humidity may indicate a broader impairment of essential anti‐*Plasmodium* and anti‐bacterial factors under ETH conditions. However, the benefit for *A. stephensi* survival conferred by the elevated temperature and relative humidity (Figure 4) seems to outweigh the potential negative effects associated with the downregulation of key immunity genes. The *E. coli‐*induced gene expression observed in adult mosquitoes contrasts with the non‐response in larvae. However, the significantly higher survival of *E. coli* ‐challenged larvae (Figure 4A) even though most innate immunity responses measured were not activated, suggests potential activity of mosquito infection tolerance mechanisms or non‐immunity‐related factors under high temperature/high humidity. It is possible that other immune pathways not measured in this study are more active in infected mosquitoes at elevated conditions, compensating for the overall reduced immunity. In addition, at higher temperature and humidity, mosquitoes may be dampening the activity of energetically demanding immune processes to conserve resources. Differences in transcriptional expression between larval and adult stages can also be related to differences in tradeoffs between immunity and development between the two stages, with larvae allocating more resources toward growth‐related mechanisms than defense.

Overall, our infection‐response studies show that the mosquito's immunity to bacterial infection under fluctuating environmental conditions is qualitatively different from that under static conditions.

## 4 Conclusion

Research on *Anopheles* innate immunity, including the identification of novel gene targets and promoters for the development of mosquito‐based malaria control strategies, is typically performed under fixed laboratorial climatic settings. These are optimal yet artificial environments; in nature, mosquitoes and the pathogens they vector do not live in static climate conditions. Here, we employed a novel experimental approach using combined fluctuating temperature and relative humidity to investigate the impact of increases in these climate parameters on the innate immunity, development, and survival of the *A. stephensi* malaria vector.

In summary, using combined variable temperature and relative humidity from the egg to the adult stage, this study's findings reveal that elevated realistic fluctuating temperature and humidity conditions:

1. Elevate *A. stephensi* basal immunity possibly to increase mosquito readiness to cope with microbial exposure.
2. Alter the transcriptional activity of promoters used to express transgenes with potential implications for the effectiveness of genetically modified mosquito strategies.
3. Overall decrease innate immune responses induced by bacterial infection. This could reduce the mosquito's ability to control *Plasmodium*, potentially increasing malaria transmission. This hypothesis will be tested in future studies.
4. Accelerate pre‐adult development and enhance mosquito survival post‐infection, suggesting faster generation turnover potentially increasing mosquito population size and malaria transmission.

## 5 Future Perspectives

Given the complex and profound impacts of environmental variability revealed by our study, research employing environmental fluctuations should become the new standard protocol in vector‐pathogen studies. This approach, which moves beyond reliance on static laboratory conditions, is crucial for accurately understanding and predicting the dynamics of vector‐borne disease transmission in a globally changing climate. It is equally critical for studies addressing the impact of long‐term climate conditions on mosquito adaptation through genetic selection.

While our study employed predicted climate change‐associated increases in temperature and humidity of a specific geographic region and time of year as a proof‐of‐principle, climate change varies according to geography and season. For instance, climate change projections for Pakistan, an endemic country for *A. stephensi*, indicate an increase in annual mean temperature slightly higher than 4°C (SSP3‐7.0) by 2100 (World Bank 2022), while projections for future precipitation vary between different studies (Ali et al. 2021; Shah and Sharifi 2025). In future studies, other scenarios and temperature/humidity variations should be explored, including extreme climatic events such as heat waves. Given *A. stephensi*'s rapid expansion into Africa and its ability to thrive in urban environments (Taylor et al. 2024), which are often characterized by higher temperatures than surrounding rural areas, future studies should also specifically explore the impact of such urban‐specific environmental conditions on its biology and vectorial capacity. These include investigating how elevated urban temperatures and associated humidity dynamics, potentially exacerbated by climate change, modulate *A. stephensi* immunity and *Plasmodium* transmission.

While this study focused on the combined effects of temperature and relative humidity—the two climatic factors that are used as static parameters in standard mosquito insectaries—the individual contribution of humidity and other environmental factors influencing mosquito immunity and vector competence should also be assessed. The minimum RH used in this study was 75%; however, real‐world diurnal relative humidity in Addis Ababa, Ethiopia, can fluctuate below 75% (NASA Power 2025). This technical limitation could have potentially biased our results by exposing control mosquitoes to higher minimum humidity levels; hence, future studies should aim for lower relative humidity settings.

In our study, larvae were maintained on a standardized laboratory diet (ground fish food supplement and cat food pellets). It is important to note that natural field settings present widely variable nutritional conditions, which can significantly impact various mosquito life‐history traits, as well as hemocyte numbers and the expression of innate immunity genes (Telang et al. 2012).

In future studies, it will be important to address the impact of elevated fluctuating temperature and relative humidity on mosquito biology at higher resolution than our proof‐of‐principle studies, using multi‐omics analyses (genome, epigenome, transcriptome, proteome and metabolome). In parallel, incorporating *Plasmodium* infection experiments to directly evaluate vector competence, including measuring oocyst rates, will be addressed in future studies to fully understand how these fluctuating environmental conditions impact *P. falciparum* transmission.

## Author Contributions

**Thais Lemos‐Silva:** formal analysis, investigation. **Emma De Neef:** formal analysis, investigation. **Yarno Valgaerts:** investigation. **Maria L. Simões:** conceptualization, formal analysis, funding acquisition, investigation, methodology, project administration, resources, supervision, writing – original draft, writing – review and editing.

## Conflicts of Interest

The authors declare no conflicts of interest.

## Supporting information


**Data S1:** gcb70382‐sup‐0001‐Data S1.pdf.

**Figure S1:** gcb70382‐sup‐0002‐FigureS1.tif.

**File S1:** gcb70382‐sup‐0003‐File S1.xlsx.

**File S2:** gcb70382‐sup‐0004‐File S2.xlsx.

**File S3:** gcb70382‐sup‐0005‐File S3.xlsx.

**Table S1:** gcb70382‐sup‐0006‐TableS1.pdf.

## Data Availability

The data that support the findings of this study are openly available in Dryad at http://datadryad.org/share/3Er12u1wC4Gpf7oJt58YTI5mOPgP8xcZZwqXc3XSnOo, reference number https://doi.org/10.5061/dryad.1vhhmgr6p.
