# Supplementary material for: Fluctuating Warm and Humid Conditions Differentially Impact Immunity and Development in the Malaria Vector Anopheles stephensi
Source: Glob Chang Biol. 2025 Aug 5;31(8):e70382. doi: 10.1111/gcb.70382 (PMC12322806; doi:10.1111/gcb.70382)
Supplement: Supplementary file 1 — Data S1: gcb70382‐sup‐0001‐Data S1.pdf. [file GCB-31-e70382-s002.pdf]

Fluctuating warm and humid conditions differentially impact immunity and development in the malaria vector *Anopheles stephensi*

Thais Lemos-Silva, Emma De Neef, Yarno Valgaerts, Maria L. Simões

Corresponding author: Maria L. Simões, mlsimoes@itg.be

Supporting Information

Supplementary Materials and Methods

Mosquito rearing and experimental design

A colony of *Anopheles stephensi* (SDA-500 strain) mosquitoes was maintained at 27°C and 75% relative humidity with a 12 h day/night cycle. Larvae were reared on ground fish food supplement and cat food pellets (Tetra). Adult mosquitoes were maintained on a 10% sucrose solution and fed on naïve blood (provided by the Red Cross-Flanders Biobank and tested for potential pathogens) through weekly standard membrane feeding, for egg production.

Bacteria challenge and enumeration studies

Escherichia coli and *Staphylococcus aureus* were grown overnight in LB broth in culture tubes in a shaking incubator at 37°C and the OD_{600} was measured (1.06 for *E. coli* and 0.83 for *S. aureus*, as in Simões et al., 2022, and Simões, Dong et al., 2017) in a cell density meter (Ultrospec 10, Amersham Biosciences). Bacteria were plated on LB agar plates and the resulting colonies were collected. For mosquito infections by thoracic pricking, a sterile needle was dipped into a thick pellet of live collected bacterial colonies. This method was chosen to ensure the transfer of high-load live bacteria directly from colonies to induce a robust challenge, consistent with previously published protocols for mosquito and *Drosophila* (Bian et al., 2005; Dimopoulos et al., 1997; Dudzic et al., 2015; He et al., 2017; Schnitger et al., 2007, 2009). The OD_{600} of the pellets resuspended in 1 ml PBS was measured (1.30 for *E. coli* and 1.27 for *S. aureus,*

corresponding to approximately 3.04 x 10^8 CFU/ml and 3.80 x 10^8 CFU/ml, respectively). Due to the nature of directly transferring solid colonies via a needle dip, the precise bacteria inoculum quantification per individual mosquito prick was not directly measured, similarly to the referenced studies. The needle used for pricking was flamed sterilized followed by cooling before every individual challenge. Survival analysis following bacterial challenge was conducted using an adaptation of our previous protocol (Simões et al., 2022; Simões, Dong et al., 2017).

For the quantification of endogenous bacteria from control and ETH mosquito midguts **(Fig. S1)**, non-blood-fed mosquitoes were surface sterilized with ethanol and rinsed with PBS, and their midguts were dissected and homogenized in PBS. Dilutions of this homogenate were plated into LB agar plates and incubated at room temperature for 3 days as in Simões et al., 2022, after which the bacterial colonies were counted and the number of colony-forming units (CFUs) of culturable bacteria was calculated.

RNA extraction and real-time qRT-PCR analysis

Following RNA extraction, cDNA was synthesized using 1 µg of total RNA with oligo(dT) primers and M-MLV reverse transcriptase (Promega), as described by the manufacturer. Quantitative analysis was performed in triplicate by quantitative real-time PCR using SYBR Green PCR Master Mix (Applied Biosystems), in a final volume of 20 µl including 1 µl diluted cDNA per well, using a StepOnePlus Real-time PCR System (Applied Biosystems). qRT-PCR cycle conditions used were as in Simões, Dong et al., 2017. For all assays, the expression levels of target genes were normalized to the levels of *An. stephensi* ribosomal *S7* gene (Dong et al., 2011). The sequences of primers used for amplification can be found in **Table S1**.

Supplementary Results and Discussion

Fluctuating elevated temperature and humidity conditions modulate the expression of melanization-regulating factors and suppress the expression of nitric oxide synthase-mediated responses upon blood feeding

In a previous study, a trend towards *NOS* increased expression was observed in *An. stephensi* (Liston strain) uninfected mosquitoes exposed to a mean ambient temperature of 32°C compared to mosquitoes exposed to 18°C (Murdock et al., 2013). In another study, *NOS* expression at 24 hours following *P. yoelii* infection peaked at 28°C compared to lower temperatures (Murdock et al., 2014). No significant changes were observed in *NOS* expression in sugar-fed mosquitoes, but significant lower expression (0.6-fold change, $p = 0.0114$) was observed in the fat body-containing carcass of blood-fed ETH mosquitoes, compared to control mosquitoes **(Fig. 2C)**

This discrepancy with previous studies may be partly attributed to our tissue-specific analysis (expression in the midgut or the fat body-containing carcass were assessed separately, whereas previous studies assessed *NOS* expression in the whole mosquito), or to a distinct role of humidity.

A recent study addressing how thermal fluctuations influence the transcriptome of blood-fed *An. stephensi* (Indian strain) mosquitoes showed that differential gene expression was to a large extent dependent on the tissue sampled, and the effect of the blood meal (Pathak et al., 2025). While Pathak et al. did not discuss specific gene signature or addressed immunity in any detail, a careful analysis of the RNAseq data identified differentially expressed putative antibacterial and anti-*Plasmodium* immune genes that we also examined in our study. Specifically, TEP1 was uniquely differentially expressed in the midguts of mosquitoes exposed to the highest temperature tested (28°C +5°C/−4°C) and in the carcasses of mosquitoes exposed to the lowest temperature tested (20°C +5°C/−4°C). Cp, Vg, PPO1 and PPO5 were differentially expressed across all three tested temperatures and enriched 24 hours after the blood meal (Pathak et al., 2025). Additionally, in another study, transcriptional profiles and the number of induced

genes varied significantly in *Drosophila melanogaster* maintained under constant or fluctuating temperatures (Sørensen et al., 2016).

Combined fluctuating higher temperature and humidity accelerate *An. stephensi* development without affecting adult lifespan

Although first instar-stage ETH larvae were larger than control ones, adults emerging at ETH conditions exhibited shorter wing lengths (2.8 mm vs. 3.3 mm average, $p < 0.0001$, **Fig. 3F**), indicative of reduced body size. Reduction in adult size at warmer temperatures has been observed in several *Anopheles* species (Agyekum et al., 2021), likely to be partly due to faster larval and pupal development limiting the time available for accumulating nutrients necessary for adult mosquito growth. Despite the smaller size of the ETH adult mosquitoes, adult lifespan remained unaffected **(Fig. 3G)**, contrasting with studies performed under constant conditions where increased temperature reduced survival in *An. stephensi* and *An. gambiae* mosquitoes (Barr et al., 2024; Singh et al., 2022).

The observed faster larvae/pupae development and unaltered adult lifespan under fluctuating elevated temperature and humidity conditions suggest that these environmental conditions may result in shorter mosquito generation times leading to larger mosquito populations, and hence potentially higher overall malaria transmission rates. However, it is an important consideration that smaller adult female mosquitoes, as observed in **Fig. 3F** for the ETH females, are often associated with reduced fecundity (Lyimo & Taken, 1993), including lower egg batch sizes, which could counterbalance some of the benefits observed. Smaller body size can also influence other key biological and epidemiological aspects, such as mating success, as larger males have a greater copulatory success than smaller males (Sawadogo et al., 2013). In addition, biting frequency is also impacted by size, as smaller females may require multiple blood meals for egg development (Takken et al., 1998). A second blood meal has been shown to shorten the *P. falciparum* extrinsic incubation period in *An. gambiae*, accelerating oocyst growth rates and causing earlier sporozoite accumulation in the salivary glands (Shaw et al., 2020), suggesting

that human malaria parasites exploit host resources provided with blood feeding to accelerate their growth (Kwon et al., 2021).

An. stephensi from the control group emerged at variable temperatures as low as 15°C, contrasting with previous observations where no *An. gambiae* adult emergence was observed at constant temperatures below 18°C (Bayoh & Lindsay, 2003), again emphasizing the need to employ realistic environmental fluctuation in mosquito studies.

Fluctuating elevated temperature and humidity prolong bacteria-challenged larval survival and adult resistance to *S. aureus*

ETH larvae displayed significantly greater resistance to PBS challenge (injury) and to systemic challenge with bacteria compared to control larvae (PBS: $p < 0.0001$; *E. coli*: $p = 0.0335$; *S. aureus*: $p = 0.0496$) **(Fig. 4A)**. While infection with *E. coli* and *S. aureus* had a slight negative impact on larval survival under high temperature high humidity conditions **(Fig. 4A)**, in agreement with previous studies focusing on the influence of temperature alone (Barr et al., 2024), the beneficial effect of elevated temperature and humidity outweighed the detrimental effects of bacterial infections.

The faster adult emergence rate **(Fig. 4B)** under ETH conditions was independent of the challenges (PBS, *E. coli* or *S. aureus*) larvae were given as it was also observed in non-challenged mosquitoes **(Fig. 3E)**. Interestingly, while unchallenged adult mosquitoes showed no difference in survival between the compared conditions **(Fig. 3G)**, ETH adult females challenged with PBS exhibited significantly higher survival ($p = 0.0424$) than control ones **(Fig. 4C)**.

Taken together, our results demonstrate that while challenged larvae benefit significantly from elevated temperature and humidity, regardless of the challenge imposed, the survival of challenged pupae is temperature- and humidity-independent, similarly to what we observed for unchallenged pupae **(Fig. 3E)**.

Fluctuating elevated temperature and humidity reveal novel immune gene expression patterns in bacteria-challenged mosquitoes

Fig. 5B findings contrast with previous studies conducted at constant temperature and humidity where *FBN9* and *TEP1* were upregulated in response to *E. coli* infection (Dong et al., 2006; Dong & Dimopoulos, 2009; League et al., 2017), while PPO1 downregulation in ETH *E.coli*-infected mosquitoes is consistent with previous findings at constant temperature and humidity (Dong et al., 2006; League et al., 2017).

While bacteria-challenged larvae and adults exhibited overall lower immune activity under ETH conditions, as a measure of most of our selected immune-marker genes, the specific gene expression responses differed. The significant downregulation of *CLIPA14* and upregulation of *CLIPA28* in adults may reflect a compensatory mechanism for the overall innate immunity suppression. The general suppression of melanization regulators in bacteria-infected ETH adults aligns with previous findings of reduced melanization at higher temperatures (Ferguson & Adamo, 2023; Martin & Hillyer, 2024; Murdock et al., 2012; Simões et al., 2022). The adult-specific upregulation of *CTL4* in response to *S. aureus* suggests a combined temperature- and humidity-sensitive adaptive role for this gene in enhancing survival of ETH mosquitoes specifically upon infection with this bacterium. These results differ from CTL4's previously described expression under static conditions (Dong et al., 2006). A future study should investigate the precise mechanism of CTL4 in the mosquito defense against *S. aureus* under fluctuating elevated temperature and humidity conditions. While most immune genes didn't show significant differences in larval expression between the ETH and control conditions, PBS-pricking-mediated stress resulted in a decrease in *Cactus* expression, which may relate to a higher Toll pathway activity to promote wound healing, as well as fighting infections with bacteria from the breeding water that may infect the larva through the wound site. Interestingly, adults showed a dynamic *FBN9* response, with the observed lower expression of this gene upon bacterial challenge **(Fig. 5B)** masking the initial priming effect (the transcriptional upregulation without pathogen

challenge) under higher temperature and humidity **(Fig. 2A)**. This observation reveals an interaction between environmental conditions and pathogen infection.

Supplementary References

Barr, J. S., Martin, L. E., Tate, A. T., & Hillyer, J. F. (2024). Warmer environmental temperature accelerates aging in mosquitoes, decreasing longevity and worsening infection outcomes. *Immunity and Ageing*, *21*(1), 61. https://doi.org/10.1186/s12979-024-00465-w

Bayoh, M. N., & Lindsay, S. W. (2003). Effect of temperature on the development of the aquatic stages of *Anopheles gambiae* sensu stricto (Diptera: Culicidae). *Bulletin of Entomological Research*, *93*(5), 375–381. https://doi.org/10.1079/BER2003259

Bian, G., Shin, S.W., Cheon, H.M., Kokoza, V. & Raikhel, A.S. (2005) Transgenic alteration of Toll immune pathway in the female mosquito *Aedes aegypti*. *Proceedings of the National Academy of Sciences of the United States of America*, **102**, 13568–13573. https://doi.org/10.1073/pnas.0502815102

Dudzic, J.P., Kondo, S., Ueda, R., Bergman, C.M. & Lemaitre, B. (2015) *Drosophila* innate immunity: regional and functional specialization of prophenoloxidases. *BMC Biology*, **13**, 81. https://doi.org/10.1186/s12915-015-0193-6

Ferguson, L. V., & Adamo, S. A. (2023). From perplexing to predictive: are we ready to forecast insect disease susceptibility in a warming world? *Journal of Experimental Biology*, *226*(4), jeb244911. https://doi.org/10.1242/jeb.244911

Kwon, H., Simões, M. L., Reynolds, R. A., Dimopoulos, G. & Smith, R. C. (2021). Additional Feeding Reveals Differences in Immune Recognition and Growth of *Plasmodium* Parasites in the Mosquito Host. *mSphere*, *31*(6), e00136-21. https://doi.org/10.1128/mSphere.00136-21

Lyimo, E. O., & Takken, W. (1993). Effects of adult body size on fecundity and the pre-gravid rate of *Anopheles gambiae* females in Tanzania. *Medical and Veterinary Entomology*, *7*(4), 328-332. https://doi.org/10.1111/j.1365-2915.1993.tb00700.x

Martin, L. E., & Hillyer, J. F. (2024). Higher temperature accelerates the aging-dependent weakening of the melanization immune response in mosquitoes. *PLOS Pathogens*, *20*(1), e1011935. https://doi.org/10.1371/journal.ppat.1011935

Murdock, C. C., Moller-Jacobs, L. L., & Thomas, M. B. (2013). Complex environmental drivers of immunity and resistance in malaria mosquitoes. *Proceedings. Biological Sciences*, *280*(1770), 20132030. https://doi.org/10.1098/rspb.2013.2030

Pathak, A. K., Quek, S., Sharma R., Shiau, J. C., Thomas, M. B., Hughes, G. L., Murdock, C. C. (2025). Thermal variation influences the transcriptome of the major malaria vector *Anopheles stephensi*. *Communications Biology,* 8(112). https://doi.org/10.1038/s42003-025-07477-2

Sawadogo, S.P., Diabaté, A., Toé, H.K., Sanon, A., Lefevre, T., Baldet, T., Gilles, J., Simard, F., Gibson, G., Sinkins, S. & Dabiré, R.K. (2013). Effects of Age and Size on *Anopheles gambiae* s.s. Male Mosquito Mating Success. *Journal of Medical Entomology*, *50*, 285–293. https://doi.org/10.1603/ME12041

Shaw, W.R., Holmdahl, I.E., Itoe, M.A., Werling, K., Marquette, M., Paton, D.G., Singh, N., Buckee, C.O., Childs, L.M. & Catteruccia, F. (2020). Multiple blood feeding in mosquitoes shortens the *Plasmodium falciparum* incubation period and increases malaria transmission potential. *PLOS Pathogens*, *16*, e1009131. https://doi.org/10.1371/journal.ppat.1009131

Singh, P., Pande, V., & Dhiman, R. C. (2022). Revising the impact of temperature on survival of *Anopheles stephensi* and *Aedes aegypti* and implications on extrinsic incubation period. *Journal of Communication Disorders*, *54*(1), 60–66. https://doi.org/10.24321/0019.5138.202251

Sørensen, J. G., Fristrup Schou, M., Kristensen, T. N., Loeschcke, V. (2016). Thermal fluctuations affect the transcriptome through mechanisms independent of average temperature. *Scientific Reports*, 6, 30975. https://doi.org/10.1038/srep30975

Takken, W., Klowden, M.J. & Chambers, G.M. (1998). Effect of Body Size on Host Seeking and Blood Meal Utilization in *Anopheles gambiae* sensu stricto (Diptera: Culicidae): the

Disadvantage of Being Small. *Journal of Medical Entomology*, *35*, 639–645. https://doi.org/10.1093/jmedent/35.5.639
