# Supplementary figures and images for: Fluctuating Warm and Humid Conditions Differentially Impact Immunity and Development in the Malaria Vector Anopheles stephensi
Source: Glob Chang Biol. 2025 Aug 5;31(8):e70382. doi: 10.1111/gcb.70382 (PMC12322806; doi:10.1111/gcb.70382)

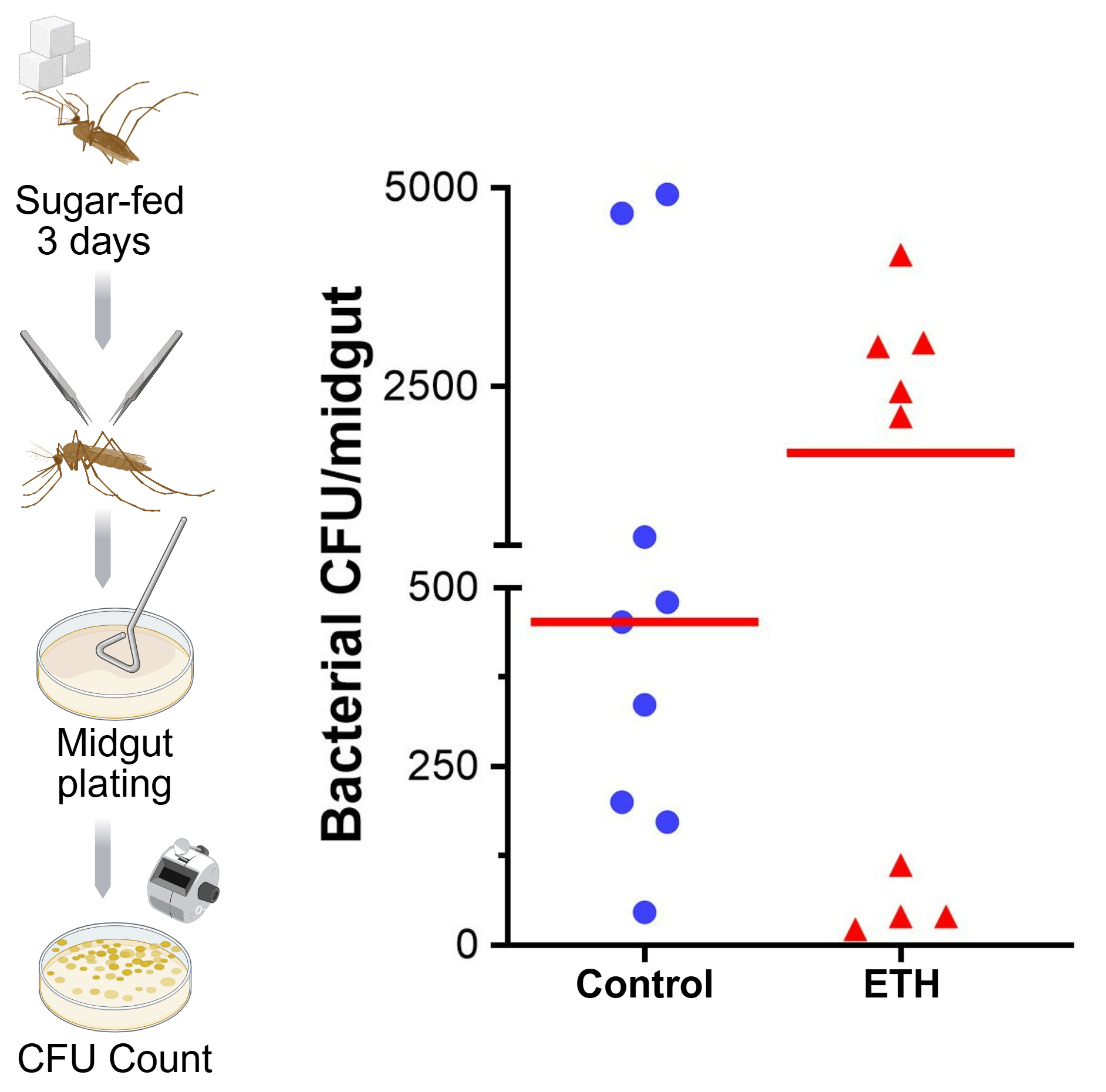

Supplement: Supplementary file 2 — Figure S1: gcb70382‐sup‐0002‐FigureS1.tif. [file GCB-31-e70382-s001.tif]
