# Supplementary material for: Fluctuating Warm and Humid Conditions Differentially Impact Immunity and Development in the Malaria Vector Anopheles stephensi
Source: Glob Chang Biol. 2025 Aug 5;31(8):e70382. doi: 10.1111/gcb.70382 (PMC12322806; doi:10.1111/gcb.70382)
Supplement: Supplementary file 6 — Table S1: gcb70382‐sup‐0006‐TableS1.pdf. [file GCB-31-e70382-s004.pdf]

Table S1. List of primers used in this study.

Gene name	Accession number	Forward primer (5'-3')	Reverse primer (5'-3')	Reference
AsAPL1C	ASTE016290	CTACAGAGCGAAATACAGCA	CAGATGTGCTATCACCTTGT	Billingsley et al., 2021
AsCactus	ASTE006481	GCTCGAGACGACTACTTACG	GCTCCACGTTTCGTTAGGTC	Mitri et al., 2020
AsCLIPA14	ASTE008190	ATGGAGCGGGCGTAATTGAT	ACAAAGTCCTTCTCGCAGCA	designed for this study
AsCLIPA28	ASTE009395	ATCCGACCCATTTGCCTACC	GTTCGCATACACTCCCCGAT	designed for this study
AsCp	ASTE010780	TTCGACTGGTACGTGTTCCC	GTAAGGTTTGCGGGTCTTGC	designed for this study
AsCTL4	ASTE002637	AGTGGTATTCGGTGGGGTTT	GCAAAACGGTTCCGAAAGCT	designed for this study
AsFBN9	ASTE008268	AACAATCTGACCGCACTGC	TGTGACGCATTCCCTGTAG	Bai et al., 2019
AsFREP1	ASTE008330	CGTCCCGGAATGAGTGGATT	CTCCGTCACGTTTGCATCAC	designed for this study
AsLRIM1	ASTE000814	GAGGAAAATGCTCGGATGAA	CGACGGCTGAACCTTACTGA	Billingsley et al., 2021
AsNOS	ASTE008593	ACATCAAGACGGAAATGGTTG	ACAGACGTAGATGTGGGCCTT	Kajla et al., 2016
AsPPO1	ASTE004215	GGTCAACTTCCTCACGCCCAAC	CCTGCCAGCATATAGACGGATAAGC	Zhu et al., 2022
AsPPO5	ASTE016295	ACCTAACCGACCAATACCGC	TCGGTGGTGTAATGTTCGGA	designed for this study
AsREL1	ASTE011378	AAACGTTCCCCAGCATACAG	CCTCGACGTCCTTCTTCTTG	Joshi et al., 2017
AsREL2	ASTE010360	GTTCCGCTTCCGCTATCAGT	CGCAACTCTACCGTGGGGAA	Billingsley et al., 2021
AsS7	ASTE004816	TCGGTTCCAAGGTGATCAAAGC	AGCGCGGTCTCTTCTGCTTGT	Dong et al., 2011
AsTEP1	ASTE016444	TTGCTGTCGTTCGTGATA	AGCGTGATGGTGTAGTCG	Billingsley et al., 2021
AsVg	ASTE003745	AACAACCGCTCTGCCTACAA	ACGACCTTGTGAGCAACGAT	designed for this study

References

Bai, L., Wang, L., Vega-Rodríguez, J., Wang, G., Wang, S. (2019). A gut symbiotic bacterium *Serratia marcescens* renders mosquito resistance to *Plasmodium* infection through activation of mosquito immune responses. *Frontiers in Microbiology, 10*. https://doi.org/10.3389/fmicb.2019.01580

Billingsley, P. F., George, K. I., Eappen, A. G., Harrell 2nd, R. A., Alford, R., Li, T., Chakravarty, S., Sim, B. K. L., Hoffman, S. L., O´Brochta, D. A. (2021). Transient knockdown of *Anopheles stephensi* LRIM1 using RNAi increases *Plasmodium falciparum* sporozoite salivary gland infections. *Malaria Journal*, *20*(1). https://doi.org/10.1186/s12936-021-03818-8

Joshi, D., Pan, X., McFadden, M. J., Bevins, D., Liang, X., Lu, P., Thiem, S., Xi, Z. (2017). The maternally inheritable Wolbachia wAlbB induces refractoriness to *Plasmodium berghei* in *Anopheles stephensi*. *Frontiers in Microbiology, 8*. https://doi.org/10.3389/fmicb.2017.00366

Kajla, M., Choudhury, T. P., Kakani, P., Gupta, K., Dhawan, R., Gupta, L., Kumar, S. Silencing of *Anopheles stephensi* heme peroxidase HPX15 activates diverse immune pathways to regulate the growth of midgut bacteria. (2016). *Frontiers in Microbiology*, ***7***. https://doi.org/110.3389/fmicb.2016.01351

Mitri, C., Bischoff, E., Eiglmeier, K., Holm, I., Dieme, C., Brito-Fravallo, E., Raz, A., Zakeri, S., Nejad, M. I. K., Djadid, N. D., Vernick, K. D., Riehle, M. M. (2020). Gene copy number and function of the APL1 immune factor changed during *Anopheles* evolution. *Parasites and Vectors*, *13*(1). https://doi.org/10.1186/s13071-019-3868-y

Zhu, F., Zheng, H., Chen, S., Zhang, K., Qin, X., Zhang, J., Iiu, T., Fan, Y., Wang, L., Li, X., Zhang, J., Xu, W. (2022). Malaria oocysts require circumsporozoite protein to evade mosquito immunity. *Nature Communications*, *13*. https://doi.org/10.1038/s41467-022-30988-z
